# Endogenous Vasoactive Peptides and Vascular Aging-Related Diseases

**DOI:** 10.1155/2022/1534470

**Published:** 2022-10-03

**Authors:** Yao Chen, Yongfen Qi, Weiwei Lu

**Affiliations:** ^1^Department of Physiology, School of Basic Medical Sciences, Chongqing Medical University, Chongqing 400016, China; ^2^Key Laboratory of Molecular Cardiovascular Science, Ministry of Education, Peking University Health Science Center, Beijing 100191, China; ^3^Department of Physiology and Neurobiology, Medical College of Soochow University, Suzhou 215123, China

## Abstract

Vascular aging is a specific type of organic aging that plays a central role in the morbidity and mortality of cardiovascular and cerebrovascular diseases among the elderly. It is essential to develop novel interventions to prevent/delay age-related vascular pathologies by targeting fundamental cellular and molecular aging processes. Endogenous vasoactive peptides are compounds formed by a group of amino acids connected by peptide chains that exert regulatory roles in intercellular interactions involved in a variety of biological and pathological processes. Emerging evidence suggests that a variety of vasoactive peptides play important roles in the occurrence and development of vascular aging and related diseases such as atherosclerosis, hypertension, vascular calcification, abdominal aortic aneurysms, and stroke. This review will summarize the cumulative roles and mechanisms of several important endogenous vasoactive peptides in vascular aging and vascular aging-related diseases. In addition, we also aim to explore the promising diagnostic function as biomarkers and the potential therapeutic application of endogenous vasoactive peptides in vascular aging-related diseases.

## 1. Introduction

Cardiovascular and cerebrovascular diseases are the most common causes of death among the elderly [1]. Many of these age-related vascular diseases, such as atherosclerosis, hypertension, vascular calcification, abdominal aortic aneurysms (AAAs), Alzheimer's disease, cerebral small vessel disease, and stroke, are mainly due to vascular aging [2, 3]. Thus, vascular aging is recently regarded as a critical factor in the prevalence and mortality of vascular disorders in older people. Effective interventions to combat vascular aging are urgent for the prevention of age-related vascular diseases.

**Figure 1:**
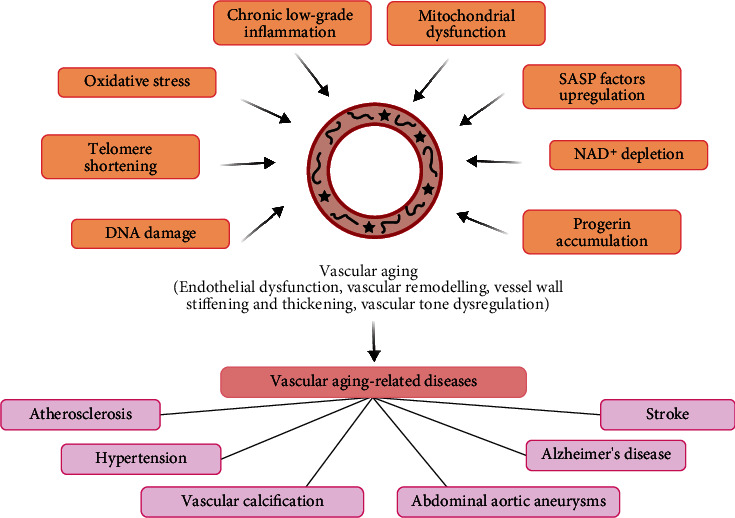
Vascular aging and related diseases. The mechanisms of vascular aging include oxidative stress, inflammation, mitochondrial dysfunction, telomere shortening, DNA damage, upregulation of SASP factors, NAD^+^ depletion, and progerin accumulation. Vascular aging is a pivotal risk factor promoting the development and progression of vascular aging-related diseases, such as atherosclerosis, hypertension, vascular calcification, abdominal aortic aneurysms, Alzheimer's disease, and stroke. Abbreviations: SASP: senescence-associated secretory phenotype.

A large body of evidence documents that some endogenous vasoactive peptides serve as novel intercellular information communicators between cells, which are associated with most of aging-related disorders [4, 5]. Vasoactive peptides are produced in neurons, heart, blood vessels, and other tissues, and they help maintain vascular homeostasis and mediate the pathogenesis of cardiovascular diseases (CVDs) mainly by regulating the functions of endothelial cells (ECs) and vascular smooth muscle cells (VSMCs) via their receptors. They have many advantages, including small molecular weight, simple structure, wide tissue distribution, diverse biological effects, rapid synthesis and metabolism, and low immunogenicity [4–6]. Recently, vasoactive peptides have attracted attention because of their value in the diagnosis and treatment of some CVDs. For example, angiotensin-converting enzyme (ACE) inhibitors and angiotensin II type 1 receptor blockers (ARBs) are primarily designed to block and/or reduce the detrimental effects of angiotensin II (Ang II); and brain natriuretic peptide (BNP) and N-terminal proBNP (NT proBNP) are used as nonspecific biomarkers for the diagnosis of heart failure. Therefore, vasoactive peptides might be a potential biomarker for diagnosis and therapeutic target in treating vascular aging-related diseases. Here, we summarize the current knowledge on the roles of some common vasoactive peptides in vascular aging and age-related vascular pathologies, as well as the diagnostic use and therapeutic application of these peptides (Figure 1).

## 2. Vascular Aging and Vascular Aging-Related Diseases

CVDs and cerebrovascular diseases are the major cause of death globally [7]. Aging is one of the main risk factors for multiple vascular disorders, including CVDs and cerebrovascular diseases, resulting in a progressive structural and functional decline of the vascular networks [3, 7]. Today, the aging population is growing dramatically, and the number of adults over 65 is projected to increase from 617.1 million to 1 billion by 2030 and to 1.6 billion by 2050 [8]. Many age-related vascular diseases are due to the aging-induced functional and phenotypic alterations of the microcirculation [2]. Accumulating evidence indicated that vascular aging is a major trigger of many vascular disorders and enhances the incidence and mortality of atherosclerosis [9], hypertension [10], cerebral small vessel disease [11], Alzheimer's disease [12], stroke [11], etc.

Vascular aging is a specific type of organic aging characterized by structural and functional alterations in the vasculature, including vascular cell senescence, extracellular matrix (ECM) remodeling, oxidative stress, inflammation, apoptosis, and calcification, which are implicated in the development and progression of vascular aging-related diseases [13]. Vascular aging and vascular diseases interact with each other. The aging vasculature provides a microenvironment for the onset and development of vascular diseases, while vascular diseases accelerate the process of vascular aging. The mechanisms involved in the pathogenesis of vascular aging include oxidative stress, mitochondrial dysfunction, inflammation, premature cellular senescence, DNA damage, maladaptation to molecular stresses, impaired maintenance of proteostasis, deregulated nutrient sensing, stem cell dysfunction in the vascular system, and impairments in the synthesis and secretion of vasoactive molecules [2, 14–16]. These molecular and physiological alterations lead to the stiffening and thickening of the vessel wall, as well as endothelial dysfunction and vascular tone dysregulation [15, 17]. ECs and VSMCs are the two primary major cell types within the vasculature, and their structural and functional disruptions are the major causes of vascular aging, which could influence the onset, process, and severity of age-related vascular diseases [2, 18]. Therefore, improving functional disorders of ECs and VSMCs by targeting fundamental cellular and molecular aging processes might ameliorate vascular aging, providing potential therapeutic strategies to prevent/delay vascular aging-related diseases (Figure 1).

Aging occurs progressively in both men and women, however, the progress of vascular aging in women appears to be slowed, as compared to men, and accelerated during the menopause transition. The reasons for these sex differences in vascular aging are not completely understood but may be related to gonadal aging and the changes of sex hormones during aging [19]. Previously, the regulatory influence of sex hormones on vascular aging was attributed to the effects of menopause and declines in estradiol in women. Increasing data suggest that decreasing testosterone levels in men contribute to accelerated vascular aging [19, 20]. To date, the effects of testosterone in women remain ambiguous. Although the androgen levels in healthy women across the lifespan show wide variations, all androgens and androgenic prohormones including testosterone, seem to decrease with age [21]. Mineralocorticoid receptors in smooth muscle cells may contribute to vascular aging in both sexes but by different mechanisms [22].

Since vascular aging is the basis of a variety of vascular diseases, better understanding of the mechanism, early detection, and intervention of vascular aging will provide a new direction of diagnosis, treatment, and prognosis of vascular aging-related diseases, especially in cardiovascular and cerebrovascular diseases. Additionally, sex-specific therapeutic strategies are also necessary for the prevention of vascular aging-related diseases.

## 3. Vasoactive Peptides and Vascular Aging-Related Diseases

### 3.1. Angiotensin 1-7 (Ang-(1-7))

Ang-(1-7), a biologically active peptide in the renin-angiotensin system (RAS) family, is converted from Ang I, Ang II, or Ang-(1-9) by neprilysin (NEP), ACE2, vascular endothelium prolyl endopeptidase, or smooth muscle thimet oligopeptidase [23]. By virtue of its actions on the G protein-coupled receptor Mas, Ang-(1-7) opposes the cardiovascular deleterious effects of Ang II, one of the most abundant components of RAS. Recent preclinical translational studies indicate a critical counterregulatory role of the ACE2/Ang-(1-7) axis on the activated RAS and other non-RAS stressors involved in many aging-related diseases.

RAS plays a central role in regulating extracellular fluid volume, vascular biology, normal blood vessel function, and pathogenesis of CVDs [23]. In the RAS, angiotensinogen is transformed by renin into Ang-I, which is then mainly cleaved into the potent vasoconstrictor, Ang-II via ACE. Ang II, the main vasoactive peptide in the systemic RAS, causes vasoconstriction and salt and water retention, induces cardiac and vascular remodeling, and promotes the detrimental effects of other neurohormonal systems, mainly via the Ang II type 1 receptor (AT1R), which is widely expressed in the cardiovascular system [24]. By binding to the Ang II type 2 receptor (AT2R), Ang II can also mediate vasodilatory effects. ACE inhibitors, ARBs, and inhibitors that target other vasopeptidases as well as receptors for vasoactive peptides are primarily designed to attenuate the adverse pathophysiological effects of Ang II for treatment of a wide range of indications related to CVDs and renal diseases [25]. These inhibitors of RAS have showed favourable long-term effects in experimental and clinical CVDs [4]. Increasing evidence demonstrated that upregulation of tissue RAS participates in the pathogenesis of vascular aging promoting intimal thickening and vascular remodeling in aged animals and old people [26, 27]. Furthermore, pharmacological inhibition of RAS activity can attenuate the stiffness of vasculature in aged animals and elderly humans independently of decreasing blood pressure [28, 29]. Recently an extended renin-angiotensin-aldosterone system, as well as the local RAS in the vasculature play a tissue-specific role in modulation of aging processes [25].

Ang (1-7) plays a counterregulatory role to ACE/Ang II/AT1R axis, eliciting a range of effects such as vasodilatation, inhibition of VSMC proliferation, and protection against oxidative stress and inflammation [24]. In vitro, Ang-(1-7) exerted protective effects against human EC senescence by consecutively activating klotho and the cytoprotective nuclear factor-erythroid 2-related factor 2 (Nrf2)/heme oxygenase-1 (HO-1) pathway through the Mas receptor [30]. Similarly, Ang-(1-7) also mitigated the senescence of VSMCs by inhibiting nicotinamide adenine dinucleotide phosphate (NADPH) oxidase and nuclear factor kappa-B (NF-*κ*B) induced by Ang II [31].

Ang-(1-7) acts as a potent vasodilator in multiple vascular beds. In spontaneously hypertensive rats (SHRs), Ang-(1-7) caused dose-dependent relaxation in the artery by activating the NO-cyclic guanosine monophosphate- (cGMP-) dependent protein kinase G (PKG) pathway via the Mas receptor [32]. In addition, Ang-(1-7) could decrease the Ang II-induced contractile response in rat aortas by inhibiting extracellular regulated protein kinase (ERK) 1/2 activation via regulation of mitogen-activated protein kinase phosphatase-1 (MKP-1) activity [33]. In aged male rats, the vasodepressor effect of Ang-(1-7) was mediated via stimulation of both AT2R and Mas receptor compared to exclusive stimulation of AT2R in adult male rats [34]. However, in old female mice, the Ang-(1-7)-mediated vasodilatory effect was absent but could be restored by estradiol replacement. Thus, the vasodilatory effect of Ang-(1-7) could be affected by estradiol changes during aging [35]. Current research has shown that Ang-(1-7) attenuated the development of atherosclerosis [36] and increased the stability of atherosclerotic plaques [37] through activation of the Mas receptor [36]. Likewise, Ang-(1-7)-treated AAA mice showed decreased vascular inflammation, ECM degradation, and VSMC apoptosis, and its effects were mediated by inhibition of ERK1/2 signaling pathway via both Mas and AT2R [38].

**Figure 2:**
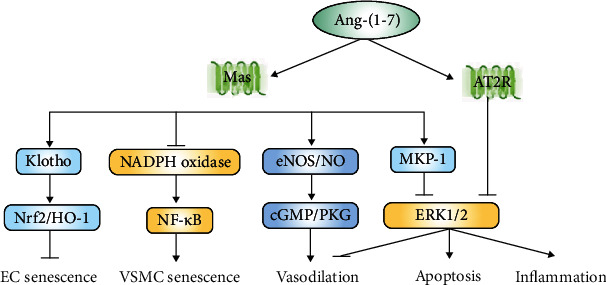
The mechanisms of Ang-(1-7) in protecting against vascular damage. Most cardiovascular effects of Ang-(1-7) are likely to be mediated by the Mas receptor. However, Ang-(1-7) also has a weak affinity for AT2R. By binding to its receptors, Ang-(1-7) exerts a wide range of effects on blood vessels, such as antisenescence, anti-inflammatory, antiapoptotic, and vasodilative effects. Abbreviations: Ang-(1-7): angiotensin-(1-7); AT2R: angiotensin type 2 receptor; cGMP: cyclic guanosine monophosphate; EC: endothelial cell; ERK: extracellular regulated protein kinases; eNOS: endogenous nitric oxide synthase; HO-1: heme oxygenase-1; MKP-1: mitogen-activated protein kinase phosphatase-1; NADPH: nicotinamide adenine dinucleotide phosphate; NF-*κB*: nuclear factor kappa-B; NO: nitric oxide; Nrf2: nuclear factor-erythroid 2-related factor 2; PKG: protein kinase G; VSMC: vascular smooth muscle cell.

Evidence from clinical studies points to a possible interaction between Ang-(1-7) and vascular lesions. Ang-(1-7) levels in plasma were significantly lower in the Alzheimer's disease patients. The compensatory circulating Ang-(1-7) might protect against Alzheimer's disease-related damages by increasing cerebral blood flow, reducing blood-brain barrier (BBB) permeability, and inhibiting inflammation [39]. Durand et al. [40] have shown that Ang-(1-7) restored physiological nitric oxide- (NO-) mediated vasodilation of atrial and adipose arterioles in humans with coronary artery disease. In particular, the protective actions of Ang-(1-7) on the human endothelium seem to occur via increased transcriptional activation of TERT, the catalytic subunit of telomerase [40]. However, when administered intravenously, Ang-(1-7) has a short bioavailability for its rapid degradation by circulating enzymes. Several stabilized forms are currently under investigation, including cyclic Ang-(1-7), cyclodextrins-included or bioencapsulated Ang-(1-7), and Ang-1-6-O-Ser-Glc-NH2 (PNA5) [41]. More clinical pharmacological strategies need to be explored in the future (Figure 2).

### 3.2. Apelin

Apelin, first discovered in 1998, is an endogenous ligand for the G protein-coupled receptor APJ [42]. The apelin-APJ signal transduction pathway is widely expressed throughout the cardiovascular system. Preproapelin, a 77-amino acid, is sequentially decomposed by ACE into several shorter C-terminal bioactive peptides, including apelin-12, apelin-13, apelin-17, and apelin-36 [43, 44]. Apelin-13 is the most potent peptide that has the primary active biological function [43]. A large number of studies indicate that apelin/APJ is involved in the regulation of renin-angiotensin-aldosterone system activity [45, 46], oxidative stress [47, 48], inflammation [49, 50], autophagy, apoptosis [51–53], and activation of stem cells [54] during the development of aging.

Studies showed that apelin-12 and apelin-13 attenuated vascular cell senescence by various pathways in vitro. Apelin-13/APJ axis improved Ang II-induced HUVEC senescence, which in turn improved cellular viability. These cellular protections were associated with reduced reactive oxygen species (ROS) production and enhanced telomerase activity via the AMP-activated protein kinase (AMPK)/sirtuin1 (SIRT1) signaling [45]. Likewise, apelin-13 effectively attenuated calcification by suppressing ROS-mediated DNA damage and improving the dysfunction of MAPKs and phosphatidylinositol 3 kinase (PI3K)/protein kinase B (Akt) in high glucose-treated VSMCs [55]. In high glucose-treated microvascular ECs, the level of apelin-12 was markedly decreased, whereas exogenous apelin-12 treatment suppressed inflammation and oxidative stress by inhibiting C-jun kinase enzyme (JNK) and p38 mitogen-activated protein kinase (MAPK) pathway [56].

Apelin and APJ receptor expression were downregulated with age in mice, and that deficiency of apelin accelerated cardiovascular aging, whereas its restoration extended the mice healthspan [57]. ACE2 is an important target of apelin action in the vasculature. Loss of apelin caused age-dependent heart dysfunction, which was associated with decreased ACE2 levels [58]. Also, apelin knockout (KO) potentiated Ang II-induced AAA formation in mice, due to the lack of apelin-mediated upregulation of ACE2 and its prosurvival effects on VSMCs [59]. Moreover, NEP is a major enzyme that metabolizes and inactivates apelin. The researchers further found treatment with a stable apelin-17 analog, which is resistant to NEP cleavage, ameliorated Ang II-mediated AAA formation [59]. Intravenous pretreatment of rats with apelin-17 also markedly reduced cerebral infarct volume after middle cerebral artery occlusion (MCAO) [60]. In addition, in rats with vascular calcification, apelin-13 significantly ameliorated aortic calcification by preventing endoplasmic reticulum stress (ERS) through activation of Akt signaling [61].

**Figure 3:**
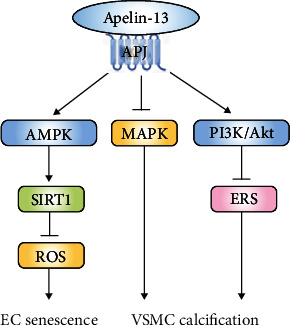
The protective effects of apelin-13 on vascular cell senescence. By binding to APJ, apelin-13 inhibits EC senescence and VSMC calcification via AMPK/SIRT1, MAPK, and PI3K/Akt pathways. Abbreviations: Akt: protein kinase B; AMPK: AMP-activated protein kinase; APJ: apelin receptor; EC: endothelial cell; ERS: endoplasmic reticulum stress; MAPK: mitogen-activated protein kinase; PI3K: phosphatidylinositol 3 kinase; ROS: reactive oxygen species; SIRT1: sirtuin1; VSMC: vascular smooth muscle cell.

In recent years, there has been growing clinical evidence of apelin's involvement in the pathogenesis of vascular aging-related diseases. A human study has demonstrated decreased plasma apelin concentrations and impaired NO bioavailability in older hypertensive patients, while high-intensity interval training reduced blood pressure by increasing apelin and nitrite/nitrate (NOx) plasma levels. Interestingly, the elevation of plasma apelin level was associated with a concomitant elevation of plasma NOx level [62]. Meanwhile, ischemic stroke patients with good collateral circulation had significantly higher plasma apelin-17 and apelin-36 levels than patients with poor collateral circulation [60]. Moreover, direct administration of the pyroglutamated form of apelin-13 has been shown to significantly increase cardiac index and lowered mean arterial pressure and peripheral vascular resistance in healthy control subjects as well as in patients with heart failure [63, 64]. However, the short half-life of apelin greatly limits its therapeutic utility for patients. Potent and safe small molecule activators of APJ need to be developed (Figure 3).

### 3.3. Calcitonin Gene-Related Peptide (CGRP) Family

CGRP family peptides, including calcitonin, CGRP, adrenomedullin (AM), intermedin (IMD), and amylin, have been identified from various vertebrates and have a range of biological activities. Accumulating evidence has suggested the protective roles of CGRP, AM, and IMD in aging-related vascular diseases. These peptides exert effects by binding to calcitonin receptor-like receptor (CRLR)/receptor activity-modifying protein (RAMP) 1, 2, or 3 receptor complexes [65].

#### 3.3.1. CGRP

CGRP is a 37-amino-acid neuropeptide that is mainly released from sensory nerve terminals and widely distributed in the central and peripheral nervous systems, as well as various organs, including the cardiovascular system [65].

CGRP exhibits antioxidant and anti-inflammatory properties in vitro. In high glucose-treated ECs, CGRP increased the NO production as well as the eNOS expression and decreased oxidative injury by inhibiting ERK1/2-NOX4 [66]. CGRP also exhibited its antioxidant effect by blocking the Src/signal transducers and activation of transduction-3 (STAT3) signaling pathway [67] and attenuated ROS-dependent apoptosis by inhibiting the calcium/calmodulin-dependent protein kinase II (CaMKII)/cAMP-responsive element-binding protein (CREB) signaling pathway in Ang II-treated VSMCs [68]. Additionally, a disintegrin and metalloproteinase domain-containing protein (ADAM17), also known as tumor necrosis factor-*α*- (TNF-*α*-) converting enzyme, was found to be involved in the effect of CGRP against VSMC inflammation though the epidermal growth factor receptor (EGFR)-ERK1/2 pathway [69].

CGRP induces vasodilation mainly by directly binding to the receptor complex formed by RAMP1 and CRLR. As compared with young controls, CGRP content was significantly decreased in the resistance arteries and plasma of middle-aged female rats. However, chronic perfusion of estradiol-17*β* to ovariectomized rats significantly enhanced CGRP and its receptor RAMP1 and thus reduced blood pressure, which suggest that the content and effect of CGRP was regulated by female sex steroid [70]. The endothelium-dependent vasodilator responses to CGRP were significantly decreased in rats during aging, which may be related to the elevation of endogenous asymmetric dimethylarginine (ADMA), an endogenous nitric oxide synthase (eNOS) inhibitor [71]. However, CGRP could also exert cardiovascular protective effects independently of NO. In a mice model of cardiovascular disease associated with endothelial dysfunction and impaired NO production, the endogenous and exogenous CGRP were able to restore blood pressure even when NO synthesis was blocked [72].

**Figure 4:**
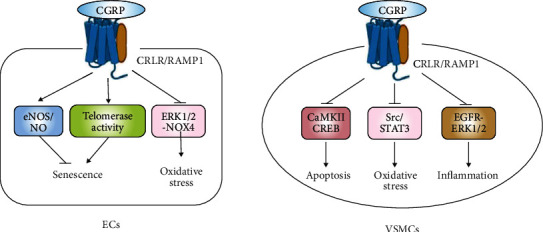
The mechanisms of CGRP in protecting against EC and VSMC injury. In ECs, CGRP improves endothelial function by increasing eNOS/NO expression, upregulating telomerase activity, and inhibiting ERK1/2-NOX4 via binding to its receptor complex CRLR/RAMP1. In VSMCs, CGRP attenuates apoptosis by inhibiting the CaMKII/CREB signaling pathway, inhibiting oxidative stress by blocking the Src/STAT3 signaling pathway, and protecting against inflammation via the EGFR-ERK1/2 pathway. Abbreviations: CGRP: calcitonin gene-related peptide; CRLR: calcitonin receptor-like receptor; RAMP: receptor activity-modifying protein; eNOS: endogenous nitric oxide synthase; NO: nitric oxide; ERK: extracellular regulated protein kinase; NOX: NADPH oxidase enzyme; CaMKII: calmodulin-dependent protein kinase II; CREB: cAMP-responsive element-binding protein; STAT3: signal transducers and activators of transduction-3; EGFR: epidermal growth factor receptor; ECs: endothelial cells; VSMCs: vascular smooth muscle cells.

CGRP has been reported to have beneficial effects in hypertensive and heart failure patients. Struthers et al. [73] infused human CGRP into normal volunteers, which caused a significant decrease in diastolic pressure and an increase in heart rate. Similarly, prolonged CGRP infusion in patients with congestive heart failure showed significant decreases in arterial pressure as well as in systemic vascular resistance and increase in cardiac output. These studies raised the possibility that CGRP is a potent endogenous vasodilator and a critical physiological regulator of hemodynamics [74]. In addition, the antihypertensive drug olmesartan could exert its vasoprotective effect in hypertensive patients partly via increased CGRP-mediated improvement of endothelial dysfunction, likely due to the increased number of circulating EPCs [75]. The effective dose of CGRP in cardiovascular-related diseases and the longer-lasting CGRP agonists require further study (Figure 4).

#### 3.3.2. Adrenomedullin (AM)

AM is a 52 amino acid multifunctional peptide discovered in 1993 that shows strong cardiovascular protective effects. High levels of AM are found in the adrenal medulla, heart, lung, and kidney. In addition, AM has been shown to circulate in plasma, and circulating AM is secreted mainly from vascular cells [76].

AM is indispensable for vascular morphogenesis during embryonic development and for postnatal regulation of blood pressure. Shindo et al. [77] showed that targeted null mutation of the AM gene was lethal to mice in utero because of abnormalities of the vascular structure. AM+/- mice survived to adulthood but upregulated blood pressure and decreased NO production, which suggest that AM exerts an effect on blood pressure via an NO-dependent pathway [77]. Similarly, Iring et al. [78] reported that the mechanosensitive cation channel PIEZO1 mediated the fluid shear stress-induced release of AM from ECs in hypertension. The released AM then activated the AM receptor/cyclic adenosine monophosphate (cAMP)/protein kinase A (PKA) signaling, which led to eNOS phosphorylation and thereby promoted NO formation to regulate vascular tone and blood pressure. AM maintains vascular homeostasis via binding to its receptor complexes CRLR/RAMP2 or CRLR/RAMP3. Most EC-specific RAMP2 KO mice (EC-RAMP2-/-) died perinatally. With aging, EC-RAMP2-/- mice showed increased oxidative stress and accelerated vascular senescence [79]. In a wire-induced vascular injury model, the RAMP2+/- vessels showed greater inflammation and oxidative stress than vessels from wild type (WT) mice [80]. AM deficiency exacerbated ischemic brain injury in old mice. The aged AM+/- mice have increased white matter damage after prolonged hypoperfusion compared to WT mice, which was related to oxidative stress induced by AM deletion [81]. In RAMP2+/- mice subjected to MCAO, recovery of cerebral blood flow was slower than in WT mice. Pathological analysis revealed a higher level of oxidative stress and less compensatory capillary growth in RAMP2+/- mice [82]. Taken together, these results indicate that the AM-RAMP2 system is a key determinant of vascular homeostasis from the prenatal stage through adulthood.

**Figure 5:**
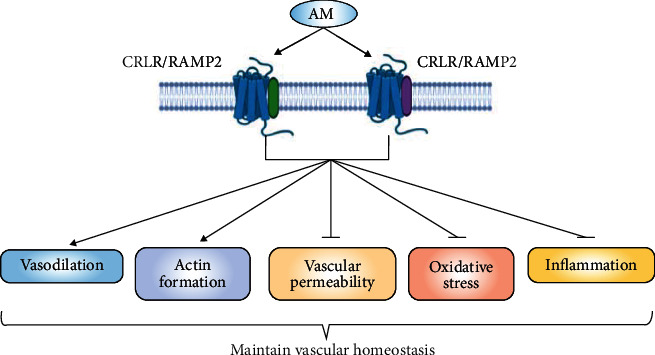
The regulatory effects of AM on vascular homeostasis. AM maintains vascular homeostasis by promoting NO and actin formation and inhibiting vascular permeability, inflammation, and oxidative stress via binding to its receptor complexes CRLR/RAMP2 or CRLR/RAMP3. Abbreviations: AM: adrenomedullin; CRLR: calcitonin receptor-like receptor; RAMP: receptor activity-modifying protein.

AM induces beneficial hemodynamic and myocardial changes in patients with heart failure. AM infusion significantly reduced mean arterial pressure and systemic vascular resistance without changing heart rate and increased cardiac output in acute heart failure patients [83]. In addition, in patients with congestive heart failure, AM also decreased mean arterial pressure and increased heart rate [84]. Age was a significant, independent variable for the plasma level of AM in the general population. The plasma level of AM increased with aging, suggesting possible relationships between the plasma AM level and age-related changes in the cardiovascular system [85] (Figure 5).

#### 3.3.3. Intermedin (IMD)

IMD is an autocrine/paracrine biologically active peptide discovered in 2004. The human IMD gene encodes a prepro-peptide of 148 amino acids. Proteolytic processing of a larger IMD precursor yields a series of biologically active C-terminal fragments, IMD_1-53_, IMD_1-47_, and IMD_8-47_. IMD_1-53_ may be the main active fragment of IMD [86]. IMD elicits its biological actions via nonselective interactions with different combinations of CRLR and the 3 RAMPs. In recent years, growing evidence has indicated that IMD is a vital bioactive peptide maintaining vascular homeostasis [86, 87].

We previously found that IMD expression was significantly decreased in old rat aortas. In addition, IMD-deficient mouse VSMCs showed senescence features coinciding with osteogenic transition compared with WT mouse VSMCs [88]. Mechanistically, the antiaging factor SIRT1 mediated the inhibitory effects of IMD on aging-associated vascular calcification [88], while in renal failure-related vascular calcification, the protective role of IMD was mainly mediated by another antiaging factor, *α*-klotho [89]. Moreover, IMD_1-53_ also inhibited the progression of atherosclerotic lesions, plaque vulnerability [90], and calcification [91] in ApoE-/- mice. Furthermore, our study demonstrated that IMD_1-53_ protected against atherosclerosis and stabilized the lesions by inhibiting ERS-C/EBP-homologous protein- (CHOP-) mediated macrophage apoptosis, and the subsequent NOD-like receptor family pyrin domain containing 3 (NLRP3) inflammasome triggered inflammation [90, 91].

**Figure 6:**
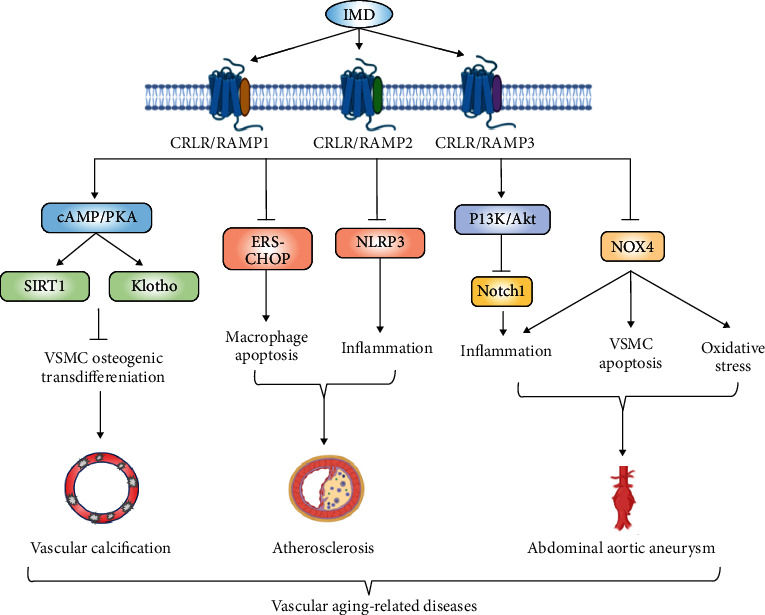
The mechanisms of IMD in attenuating vascular aging-related diseases. By binding to CRLR and three RAMPs nonselectively, IMD inhibits VSMC osteogenic transdifferentiation, macrophage and VSMC apoptosis, oxidative stress, and inflammation via different signaling pathways, such as cAMP/PKA, ERS-CHOP, and PI3K/Akt, and thus protects against vascular aging-related diseases. Abbreviations: IMD: intermedin; CRLR: calcitonin receptor-like receptor; RAMP: receptor activity-modifying protein; cAMP: cyclic adenosine monophosphate; PKA: protein kinase A; SIRT1: sirtuin1; VSMC: vascular smooth muscle cell; ERS: endoplasmic reticulum stress; CHOP: C/EBP-homologous protein; NLRP3: NOD-like receptor family pyrin domain containing 3; PI3K: phosphatidylinositol 3 kinase; Akt: protein kinase B; NOX4: NADPH oxidase enzyme 4.

In an Ang II-induced ApoE-/- mouse model of AAA, IMD_1-53_ significantly reduced the incidence of AAAs. Mechanistically, IMD_1-53_ attenuated oxidative stress and subsequent inflammation and VSMC apoptosis by inhibiting NOX4, a ROS generating NADPH oxidase enzyme [92]. In addition, we found that inhibition of Notch1-mediated inflammation by IMD could also be involved in its protection against AAAs [93]. Meanwhile, Li et al. [94] reported that IMD attenuated Ang II-induced vascular collagen remodeling by inhibiting the phosphorylation of Akt and MAPK. Although IMD is considered to be a potent cardiovascular protective factor, there are still challenges to promote the clinical application of IMD. The details of the biological effect of different IMD fragments and the enzymes responsible for its degradation are still to be defined (Figure 6).

### 3.4. C-Type Natriuretic Peptide (CNP)

CNP, the third member of the natriuretic peptide family, is a potent vasorelaxative peptide of 22 amino acids. As an autocrine and paracrine mediator, CNP can be produced and secreted by vascular ECs, VSMCs, and fibroblasts and governs a wide range of cardiovascular effects [95, 96].

CNP exerts its regulatory effects on the cardiovascular system via two cognate receptors, natriuretic peptide receptor B (NPR-B, also called guanylate cyclase B (GC-B)) and NPR-C, which induce activation of distinct signaling pathways. CNP stabilized resident perivascular mast cells at baseline and prevented their excessive activation via GC-B/cGMP signaling in mice after acute ischemia-reperfusion [97]. In addition, NPR-C in ECs mediated the role of CNP in angiogenesis and vascular remodeling in response to ischemia. These effects of CNP/NPR-C on ischemia were dependent on the activation of ERK1/2 and PI3K/Akt signaling pathways [98]. Besides, the CNP system plays a crucial role in regulating blood pressure. The vasodilatory effects of CNP on the microvasculature were endothelium-independent, which were mediated by GC-B/cGMP signaling in VSMCs of distal arterioles and capillary pericytes. These data indicated that the GC-B receptor in VSMCs and pericytes is essential for CNP to maintain normal microvascular resistance and blood pressure [99]. However, another research reported that VSMC-specific GC-B KO mice exhibited systemic blood pressure similar to control mice [100]. The mechanism of CNP/GC-B system in regulating blood pressure remains to be confirmed. Moyes et al. [96] found that administration of small-molecule NPR-C agonists promoted vasorelaxation of isolated resistance arteries and reduced blood pressure in WT animals. Treatment with CNP effectively attenuated vascular remodeling and reduced systolic blood pressure in hypertensive rats and decreased both proinflammatory and profibrotic cytokines [101]. In addition, CNP activated the vascular NO system and exerted an antioxidant effect in the aortic tissue of both hypertensive rats and normotensive rats [101]. Impaired local endogenous CNP may be associated with increased vascular calcium deposition in rat aortas with vascular calcification [102]. Administration of CNP greatly reduced vascular calcification by preventing the osteogenic transition of contractile VSMCs [102]. These results show that CNP not only regulates blood pressure but also attenuates vascular damage.

**Figure 7:**
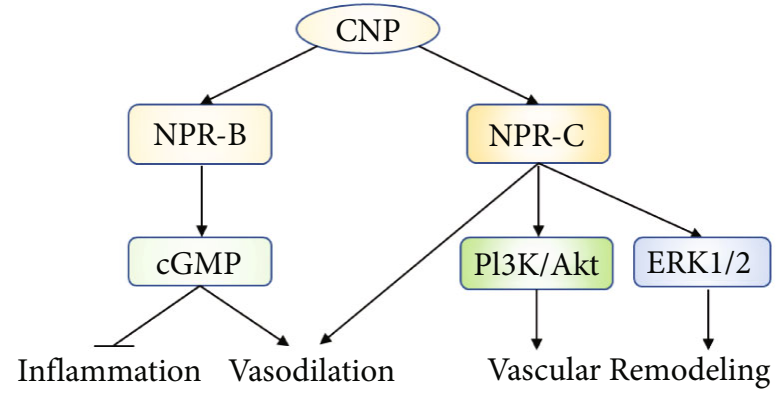
The vascular roles of CNP. CNP inhibits vascular inflammation and promotes vascular remodeling and vasodilation via its receptors NPR-B or NPR-C. Abbreviations: Akt: protein kinase B; cGMP; cyclic guanosine monophosphate; CNP: C-type natriuretic peptide; ERK: extracellular regulated protein kinases; NPR: natriuretic peptide receptor; PI3K: phosphatidylinositol 3 kinase.

A couple of clinical studies have measured plasma CNP level as a prognostic biomarker for CVDs [103–105]. Bubb et al. [98] revealed that vascular ischemia was associated with reduced levels of CNP and NPR-C, which suggest that CNP/NPR-C signaling might be critical to a proangiogenic host defense response to ischemia. In old patients with heart failure, cardiac and systemic CNP levels were increased and related to clinical severity [104]. In addition, another research found that highest concentrations of CNP identified a high-risk phenotype that included cardiovascular comorbidities and left ventricle dysfunction [106]. However, the half-life of CNP is short and circulating levels of CNP are very low. The N-terminal part (NT) of proCNP (NT-proCNP) can be more easily measured compared with CNP. Prickett et al. [105] showed that NT-proCNP largely reflected vascular integrity and was an independent predictor of both mortality and cardiac readmission in individuals with unstable angina. Taken together, targeting the CNP and its receptor pathways may therefore provide a tangible pharmacological approach to improve cardiovascular disorders (Figure 7).

### 3.5. Cortistatin (CST)

CST, a recently detected endogenous bioactive peptide, is highly expressed in the cerebral cortex and exerts an inhibitory effect on cortical activity [107]. CST and its receptors are widely distributed not only in the central nervous system, endocrine system, and immune system but also in the cardiovascular system. CST can activate somatostatin receptors (SSTRs), growth hormone secretagogue receptor 1a (GHSR1a), or the Mas-related gene X-2 receptor (MrgX2) to exert biological effects [107].

In vitro, CST inhibited the osteogenic differentiation of VSMCs in induced calcification by mitigating ERS, as shown by decreased protein expression of glucose-regulated protein 94 (GRP94) and CHOP [108], as well as the glycogen synthase kinase 3*β* (GSK3*β*)/*β*-catenin and PKC signaling pathways [109]. CST could ameliorate Ang II-stimulated VSMC proliferation and migration by inhibiting autophagy through its receptors SSTR3 and SSTR5 [110] and by inhibiting the MAPK family, including ERK1/2, p38 MAPK, JNK, and ERK5 pathways [111]. These results indicate that CST could play a critical role in regulating VSMC function under pathological conditions.

In vivo, CST also plays an important role in vascular homeostasis. Exogenous CST remarkably attenuated neointimal formation, while CST-deficient mice developed higher neointimal hyperplasic lesions than WT mice [112]. It was reported that CST treatment significantly improved hemodynamic values and arterial compliance in rats with vascular calcification, with decreased calcium deposition, alkaline phosphatase (ALP) activity, and type III sodium-dependent phosphate cotransporter-1 (Pit-1) level in aortic tissues. These effects were mediated by GHSR1a rather than SSTRs or MrgX2 [113]. Treatment with CST reduced the number and size of atherosclerotic plaques and inflammatory responses in atherosclerosis in mice. Mechanistically, CST reduced the capacity of ECs to bind and recruit immune cells to the plaques and enhanced cholesterol efflux from macrophages, thus inhibiting the formation of foam cells [114]. Additionally, CST administration significantly suppressed the incidence and severity of AAA formation in mice by alleviating the inflammatory cytokines and ROS levels, expression of matrix metalloproteinase (MMP)2 and MMP9, and cell apoptosis. Mechanistic studies showed that the protective effects of CST in AAAs were related to the blocking of ERK1/2 signaling pathways [115].

**Figure 8:**
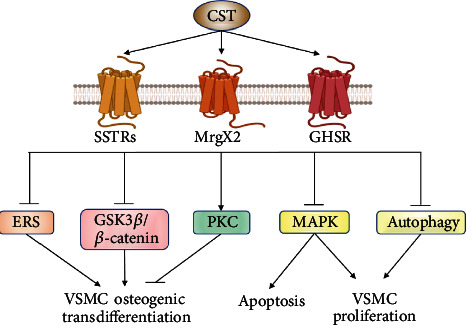
The mechanisms of CST in attenuating vascular dysfunction. CST activates three kinds of receptor including SSTRs, GHSR, and MrgX2 to exert different biological effects such as inhibit VSMC osteogenic transdifferentiation, proliferation, or apoptosis. Abbreviations: CST: cortistatin; ERS: endoplasmic reticulum stress; GHSR: growth hormone secretagogue receptor; GSK3*β*: glycogen synthase kinase 3*β*; MAPK: mitogen-activated protein kinase; MrgX2: Mas-related gene X-2 receptor; PKC: protein kinase C; SSTR: somatostatin receptor; VSMC: vascular smooth muscle cell.

At present, increasing clinical evidence indicated the potential role of CST in the regulation of CVDs. The circulating CST level was significantly increased in patients with coronary heart disease [112]. Duran-Prado et al. [112] demonstrated that VSMCs from human atherosclerotic plaques highly expressed CST, which indicates that CST could play an important role in atherosclerosis in human. However, there is still a long way to extend CST to clinical treatment (Figure 8).

### 3.6. Ghrelin

Ghrelin is a 28 amino acid peptide and functions as a natural endogenous ligand for GHSR. Ghrelin is produced by the stomach, heart, lung, and kidney and has extensive physiological effects, such as stimulating growth hormone secretion, promoting food intake, and maintaining energy homeostasis. Emerging evidence has shown favorable effects of ghrelin on cardiovascular function via either growth hormone-dependent or -independent mechanisms [116, 117].

The plasma concentration of ghrelin significantly elevated in aged mice, but exogenous administration of ghrelin failed to prolong survival in klotho-deficient mice, a model of accelerated aging, suggesting the existence of ghrelin resistance in aging process [118]. However, rikkunshito and atractylodin, two ghrelin signaling potentiators, markedly attenuated calcification and focal atrophy of cardiac muscle and prolonged survival in three mouse models of aging via the GHSR-cAMP-CREB-SIRT1 pathway [118].

Emerging evidence has shown favorable effects of ghrelin on cardiovascular function [116, 117]. Ghrelin rescued the activity of SIRT1 and the expression of superoxide dismutase-2 (SOD-2), a primary enzymatic antioxidant defense against ROS production, and resulted in great attenuation of EC senescence in mice [119]. Ghrelin could inhibit VSMC calcification by downregulating receptor activator of nuclear factor kappa B ligand (RANKL) level and increasing osteoprotegerin (OPG) expression [120] and by improving autophagy through AMPK activation [121]. In addition, ghrelin overexpression prevented high-fat diet-induced lipid accumulation in vessels by decreasing oxidative stress and thus results in improved vascular function and reduced plaque formation [122]. Furthermore, ghrelin decreased the expression of vascular endothelial growth factor (VEGF) and VEGF receptor 2 (VEGFR2) and reduced monocyte chemotactic protein 1 (MCP-1) expression, likely contributing to its protective effect in neovascularization and plaque vulnerability at an advanced stage of atherosclerosis [123].

**Figure 9:**
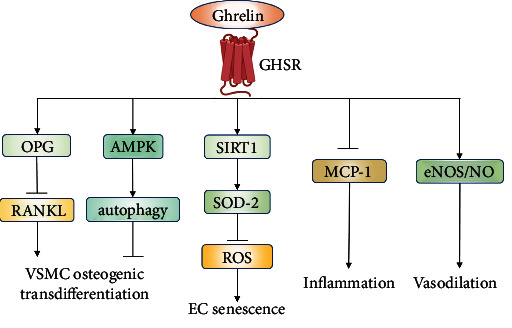
The protective effects of ghrelin on vascular aging. Ghrelin attenuates vascular calcification, inflammation, and senescence by multiple pathways through combination with the receptor GHSR. Abbreviations: AMPK: AMP-activated protein kinase; EC: endothelial cell; eNOS: endogenous nitric oxide synthase; GHSR: growth hormone secretagogue receptor; MCP-1: monocyte chemotactic protein 1; NO: nitric oxide; OPG: osteoprotegerin; RANKL: receptor activator of nuclear factor kappa B ligand; ROS: reactive oxygen species; SIRT1: sirtuin1; SOD-2: superoxide dismutase-2; VSMC: vascular smooth muscle cell.

A clinical study documented a significant negative association between ghrelin and carotid artery intima-media thickness in females with the metabolic syndrome, which suggest its active involvement in prevention of the atherosclerosis [124]. Besides, the serum ghrelin level may be a predictor of diabetic vascular calcification, because there was a negative correlation between calcium content and the ghrelin level in diabetic patient serum [120]. Moreover, exogenous ghrelin intra-arterial infusion increased endogenous antioxidant capacity, decreased levels of lipoperoxide, malondialdehyde, and phosphorylated p47, and restored the NO availability in small vessels from hypertensive patients [125]. Collectively, ghrelin may be beneficial for the restoration of impaired cardiovascular function associated with aging (Figure 9).

### 3.7. Glucagon-Like Peptide-1 (GLP-1)

GLP-1 is an incretin hormone that was first discovered as a regulator of insulin release from *β*-cells in the pancreas. As a hormone mainly secreted postprandially by intestinal L-cells, GLP-1 has multiple functions, from the regulation of insulin secretion to the control of glucose homeostasis and modulation of autonomic nervous system activity [126, 127]. GLP-1 exerts its effects through binding with GLP-1 receptor (GLP-1R), which is widely expressed in islet cells, lung, brain, kidney, and cardiovascular systems [127]. Currently, it is well acknowledged that GLP-1 exerts cardiovascular protective effects, such as alleviation of myocardial infarction [128], relaxation of vascular smooth muscle [129–131], improvement of endothelial dysfunction [132, 133], and inhibition of vascular senescence [130, 132, 134].

GLP-1/GLP-1R system may play an important role in VSMC senescence. GLP-1 treatment led to a decreasing expression of senescence markers in VSMCs treated with doxorubicin, a chemotherapeutic agent used in cancer treatment, which suggests that GLP-1 is a promising candidate for patients suffering from late doxorubicin cardiovascular toxicity [130]. Pretreatment with exendin-4, a specific ligand for GLP-1R, significantly attenuated Ang II-induced generation of H_2_O_2_ and inhibited expression of p53 and p21 and subsequent VSMC senescence by inhibiting Rac1 activation via a cAMP/PKA-dependent pathway. However, these effects were reversed in the presence of exendin (9-39), a GLP-1R antagonist [135]. Similarly, Zhou et al. [136] also demonstrated that exendin-4 could attenuate Ang II-induced ROS production and VSMC senescence. Mechanistically, exendin-4 increased the acetylation of Nrf2 and the recruitment of the transcriptional coactivator CREB binding protein (CBP) to Nrf2, which contributed to the upregulation of the antioxidant genes HO-1 and NADPH quinone oxidoreductase-1 (NQO-1) [136]. Dulaglutide, a specific GLP-1R agonist, significantly inhibited oxidized low-density lipoprotein- (Ox-LDL-) induced oxidative stress, mitochondrial dysfunction, and proinflammatory cytokine expression by increasing Krüppel-like factor 2 (KLF2) in ECs [137].

In vivo, exendin-4 had a strong dilatory effect on cortical arterioles and effectively increased cerebral blood flow via the cAMP/PKA signaling pathway in ischemic rats [138]. Exendin-4 also ameliorated BBB breakdown, reduced the infarct area, and decreased inflammation by reducing astrocyte-derived VEGF-A expression through Janus kinase 2 (JAK2)/STAT3 signaling in rat ischemic brain tissues [139]. Dipeptidyl-peptidase (DPP-4) is an enzyme that degrades native GLP-1. Mice that underwent ischemic surgery showed increased levels of DPP-4 but decreased levels of GLP-1 and antiaging factors in ischemic vessels [134]. However, DPP-4 inhibition or GLP-1R activation attenuated aortic senescence and promoted neovascularization via the upregulation of adiponectin/AdopR1 and PPAR-*γ*/PGC-1*α* signaling [134]. Diabetic rats that received exenatide, a GLP-1 analog, or sitagliptin, a DPP-4 inhibitor, showed a significant elevation of aortic endothelial function but reduced oxidative stress and inflammatory cytokine levels [140]. Notably, inhibition of RhoA/Rho associated coiled coil forming protein kinase (ROCK)/NF-*κ*B/I*κ*B *α* signaling pathway and activation of AMPK may also contribute to the protective effects of GLP-1 on aortic endothelium in diabetes [140]. Consistently, Taguchi et al. [129] showed that GLP-1 significantly enhanced endothelial-dependent relaxation in diabetic aortas, which could be mediated through activation of the AMPK/Akt pathway. In addition, GLP-1 reverses Ang II-induced vascular remodeling by decreasing expression of MMP1 through inhibition of the ERK1/2-NF-*κ*B signaling [131]. Treatment with alogliptin, a DPP4 inhibitor, or liraglutide, a GLP-1R agonist, decreased blood pressure, vascular wall thickness, and vascular collagen levels in SHRs [131]. Liraglutide treatment also alleviated vascular fibrosis, inflammation, oxidative stress, and endothelial dysfunction via GLP-1R in hypertension [133].

**Figure 10:**
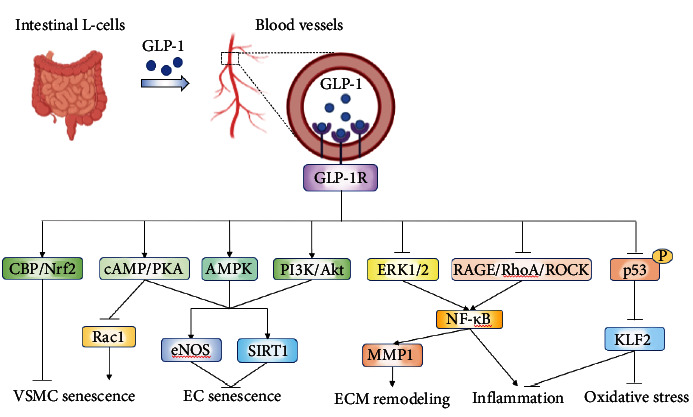
The mechanisms of GLP-1 in protecting against vascular aging. GLP-1 is mainly secreted from intestinal L-cells and exerts its vascular protective effects by binding to its receptor GLP-1R expressed in the vessels. GLP-1 attenuated VSMC senescence, EC senescence, ECM remodeling, inflammation, and oxidative stress via activating multiple signaling pathways. Abbreviations: Akt: protein kinase B; AMPK: AMP-activated protein kinase; cAMP: cyclic adenosine monophosphate; CBP: CREB binding protein; EC: endothelial cell; ERK: extracellular regulated protein kinase; ECM: extracellular matrix; eNOS: endogenous nitric oxide synthase; GLP-1: glucagon-like peptide-1; KLF2: Krüppel-like factor 2; MMP: matrix metalloproteinase; NF-*κ*B: nuclear factor kappa-B; Nrf2: nuclear factor-erythroid 2-related factor 2; PI3K: phosphatidylinositol 3 kinase; PKA: protein kinase A; RAGE: receptor for advanced glycation end products; ROCK: Rho associated coiled coil forming protein kinase; SIRT1: sirtuin1; VSMC: vascular smooth muscle cell.

Currently, the therapeutic drugs targeting the GLP-1 system have been widely used in clinical practice. GLP-1 receptor agonists (i.e., exenatide, liraglutide, semaglutide, and lalutide) and DPP-4 inhibitors (i.e., saxagliptin, sitagliptin, vigliptin, and liragliptin) have become fundamental treatment options for type 2 diabetes. Clinical studies showed that the potential cardiovascular protective effects of GLP-1 can be attributed to decreased blood pressure and direct effects on vascular cells. A research in patients with type 2 diabetes mellitus and impaired renal function showed that liraglutide decreased systemic vascular resistance and exerted vasodilatory effect in earlier chronic kidney disease [141]. The addition of semaglutide to standard of care was associated with a decrease in 10-year cardiovascular disease risk [142]. The elderly diabetic patient population treated with GLP-1 showed significant in reduction of vascular inflammation and blood glucose, as well as improvement of vascular endothelial function [143]. All these results indicate that GLP-1 participates in the regulation of vascular aging-related diseases and GLP-1R agonists could be recommended for people with diabetes and high cardiovascular risks (Figure 10).

### 3.8. Humanin (HN)

HN is the first identified mitochondrial-derived antiapoptotic peptide that is best known for its ability to suppress neuronal cell death caused by Alzheimer's disease-specific insults, including both amyloid-*β* peptides and familial Alzheimer's disease-causative genes [144]. HN acts as a ligand for two different types of receptors: the seven-transmembrane G protein-coupled receptor formyl peptide receptor-like 1 (FPRL1) and a trimeric receptor consisting of the ciliary neurotrophic factor receptor (CNTFR), the cytokine receptor WSX-1, and the transmembrane glycoprotein 130 (gp130) (CNTFR/WSX-1/gp130). In recent years, a large number of studies have revealed the beneficial properties of HN and its analog S14G-HN (HNG), including antioxidative stress, anti-inflammatory responses, and antiapoptotic effects, in age-related disorders [145].

Early studies demonstrated that HN is expressed in the EC layer of the vascular wall [146, 147] but not in the VSMC layer [146]. Therefore, in vitro studies on HN are mostly focused on ECs. In high glucose-treated HUVECs, HN increased the expression of KLF2 as well as its target gene eNOS but inhibited the secretion of TNF-*α*, IL-1*β*, vascular cell adhesion molecule-1 (VCAM-1), and E-selectin and the attachment of the monocyte cells to HUVECs. However, knockdown of KLF2 abolished these effects [148]. Thus, KLF2 may play a critical role in mediating the protective effects of HN in endothelial function. In addition, HN inhibited the activation of the NLRP3 inflammasome and endothelial inflammation via AMPK pathway and suppressed oxidative stress by downregulating the expression of NOX2 induced by free fatty acids in ECs [149]. Exogenous addition of HN to EC cultures was shown to be protective against Ox-LDL-induced apoptosis in response to oxidative stress [146]. The effect of HN on reducing lipid aggregation and EC apoptosis could be depicted in a lectin-like Ox-LDL receptor-1 (LOX-1-) dependent manner [150]. Furthermore, HNG restored the activity of the Ox-LDL-induced damaged lysosomal enzyme cathepsin D, promoting autophagic degradation of Ox-LDL in HUVECs, which may be mediated by its membrane protein receptor FPRL1 [151]. HNG also protected cell survival and suppressed inflammatory cytokines induced by oxygen deprivation via inhibition of the NF-*κ*B pathway in mouse brain ECs [152]. Although it was considered that HN was not expressed in VSMCs, HN did protect human cerebrovascular SMCs against amyloid *β*-protein-induced cell toxicity, with diminishing degradation of *α*-actin and cell death [153].

HN also attenuated microvascular remodeling, inflammation, and apoptosis in ApoE-/- mice [154]. In a murine MCAO stroke model, HNG ameliorated cerebral infarction and suppressed the production of inflammatory cytokines [152]. Furthermore, HN analogue HNGF6A prevented endothelial dysfunction and potently delayed progression of atherosclerosis in ApoE-/- mice fed on a high cholesterol diet. These effects were associated with antioxidative stress, antiapoptosis, and increased eNOS expression [147].

**Figure 11:**
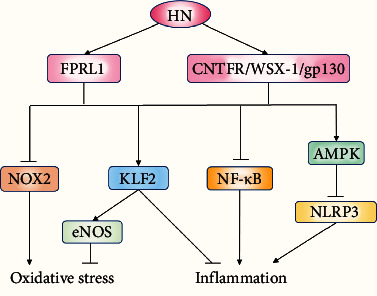
The beneficial properties of HN in vascular cell damage. HN exerts anti-inflammatory, antioxidative stress, and antiapoptosis effects via its receptors FPRL1 and the complex CNTFR/WSX-1/gp130. The target molecules of HN include KLF2, NOX2, and NLRP3. Abbreviations: AMPK: AMP-activated protein kinase; CNTFR: ciliary neurotrophic factor receptor; eNOS: endogenous nitric oxide synthase; FPRL1: formyl peptide receptor-like 1; gp130: glycoprotein 130; HN: humanin; KLF2: Krüppel-like factor 2; NF-*κ*B: nuclear factor kappa-B; NLRP3: NOD-like receptor family pyrin domain containing 3; NOX2: NADPH oxidase enzyme 2.

Circulating HN level significantly decreased in patients with coronary endothelial dysfunction, while it was positively correlated with improved coronary blood flow [155]. Besides, the improvement of cognitive function in patients with vascular dementia may be related to elevated serum HN levels [156]. Furthermore, Muzumdar et al. [157] demonstrated the circulating level of HN was decreased with age in Alzheimer's disease and type 2 diabetes mellitus patients. In contrast, a recent study [158], in which plasma HN concentrations were evaluated in 693 volunteers of different ages, demonstrated that plasma levels of HN were positively correlated with age in humans and become maximal in centenarians. These studies are consistent with the concept that HN could be considered as a marker of biological age (Figure 11).

## 4. Endogenous Vasoactive Peptide-Based Diagnostic Tools and Intervention Measures in Vascular Aging-Related Diseases

Endogenous vasoactive peptides are a serial of dynamical molecules in modulation of cardiovascular and body fluid homeostasis, providing potential intervention targets for vascular aging-related diseases. Despite intensive study of vasoactive factors at the molecular level, there are still lack of effective pharmacological approach to manipulate these systems. The main promise of vasoactive peptides as diagnostic and therapeutic tools are their property of changing dynamically with diseases and possess a wide range of biological effects. Recent studies showed significantly altered expression profiles of peptides in the process of vascular aging and strongly suggest that a decline in antivascular aging peptides may be associated with the progression of age-related vascular diseases [39, 98, 155]. As specific antibodies against these peptides have been developed, their plasma levels could be quantified by radioimmunoassay and enzyme-linked immunosorbent assay (ELISA). Additionally, variously combined use of a panel of peptides may increase the diagnostic accuracy in patients of vascular diseases.

**Figure 12:**
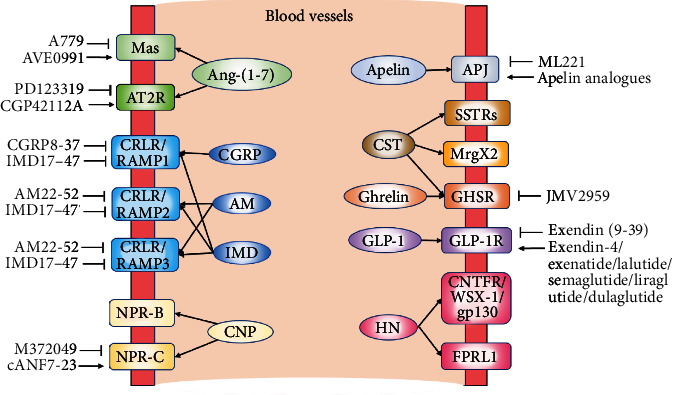
Some common vasoactive peptides and their receptors, agonists, and antagonists. Abbreviations: AM: adrenomedullin; Ang-(1-7): angiotensin-(1-7); APJ: apelin receptor; AT2R: angiotensin type 2 receptor; CGRP: calcitonin gene-related peptide; CNTFR: ciliary neurotrophic factor receptor; CNP: C-type natriuretic peptide; CRLR: calcitonin receptor-like receptor; CST: cortistatin; FPRL1: formyl peptide receptor-like 1; GHSR: growth hormone secretagogue receptor; GLP-1: glucagon-like peptide-1; gp130: glycoprotein 130; HN: humanin; IMD: intermedin; MrgX2: Mas-related gene X-2 receptor; NPR: natriuretic peptide receptor; RAMP: receptor activity-modifying protein; SSTR: somatostatin receptor.

In addition to being used as potential biomarkers, endogenous vasoactive peptides could also be served as a therapeutic tool for treatment of vascular diseases. So far, the RAS is the best studied vasoactive system, and the altered expression of its components may contribute to ACE/Ang II/AT1R-mediated pathogenesis of vascular diseases. Clinical data have shown beneficial effects in CVD patients treated with ARBs or ACE inhibitors. Similarly, drugs that target fundamental aging processes, such as endothelia dysfunction, VSMC proliferation, oxidative stress, and inflammation, may have the potential to prevent/delay a range of vascular pathologies and other age-related diseases simultaneously. Recently a variety of vasoactive peptides have emerged from basic research and translational studies that may target aging processes. As indicated in Table 1, many vasoactive peptides show anti-inflammatory and antioxidant effects, suppress VSMC proliferation, and promote endothelial NO production. Thus, the interventions with these peptides can be applied for prevention of age-related vascular diseases, and much work is needed to develop drugs that target multiple vasoactive pathways controlling vascular aging. Additionally, the peptide mimics and their receptor agonist/antagonists (Figure 12) should be studied in more depth in prospective clinical research in elderly patients with vascular disorders.

Currently, clinical studies revealed that major challenges faced by those vasoactive peptides are due to their rapid turnover in vivo and low efficiency for passing through the BBB. Thus, extensive effort is still required to overcome those unfavorable factors and to develop drug candidates with more efficiency, persistency, and safety. Moreover, development of new drug delivery system to penetrate the BBB and to continuously release effective peptide is also a prospective strategy.

## 5. Conclusions

Endogenous vasoactive peptides are now recognized as having prominent cardiovascular distribution and various modulatory effects on vascular aging-related diseases. Altered levels of these peptides in circulating blood and/or cardiovascular tissues are closely linked to age-related vascular diseases. Moreover, emerging animal studies indicate that these peptides are actively involved in the modulation of oxidative stress, inflammation, apoptosis, and vascular remodeling during aging. Thus, endogenous vasoactive peptides, alone or in various combinations, are candidate biomarkers for predicting CVDs, which may be useful for earlier detection. Vasoactive peptides, their receptors, and the proteases that degrade the same peptides may become special targets of new specific pharmacologic approaches. However, there is still an urgent need for persuasive statistical evidence in animals and clinical trials as well as site-specific studies to elucidate the generalized and precise mechanisms underlying the effects of different vasoactive peptides in vascular aging and to develop effective and safe treatments for age-related vascular diseases.

## 6. Future Research Directions

In recent years, quantitative peptidomic studies are widely used for peptide investigation with the development of mass spectrometry technology. The emergence of vasoactive peptides indicated a new perspective toward exploring the pathogenesis of vascular aging-related diseases. And significant progress has been achieved in studying the effects of vasoactive peptides on aging-induced alterations in vascular function and phenotype and vascular disorders; however, only a limited number of vasoactive peptides have demonstrated significant diagnostic and/or therapeutic impact. Hence, research efforts should continue to develop innovative strategies based on recent achievements in the biology of vascular aging, as well as in properties and effects of vasoactive peptides, to improve the diagnosis and treatment of aging-related vascular diseases. Moreover, deeper insights into the pathophysiology of vascular aging and the interaction of processes of aging and vascular diseases may led to the discovery of novel molecular targets for the peptides. The research field that is likely to solve problems concerning the mechanism of effects, control of release, and significance of a wide range of endogenous peptides in cardiovascular regulation and pathogenesis of vascular aging needs to be explored further. The role of specific signal transduction pathways mediated by these vasoactive peptides in the process of vascular aging should be better elucidated, which may intensify pharmacological drugs that interfere with the peptides net system at multiple sites. In future, studies using bioinformatics technology like single-cell gene expression analysis should also lead to improved understanding of cellular and functional heterogeneity in regulation of vascular aging by these peptides. Researchers should perform more site-specific studies to elucidate the precise mechanisms of the peptides in vascular diseases. Finally, better joint research of preclinical studies on vascular aging and human investigations is also urgently needed to yield consistent incremental clinical benefits of endogenous vasoactive peptides.

## Figures and Tables

**Table 1:** Summary of vascular protective effects and underlying mechanisms of some common vasoactive peptides.

Peptides	Receptors	Study sources	Effects and mechanisms	References
Ang-(1-7)	Mas; AT2R	In vitro studies	Anti-EC senescence through activating the cytoprotective Nrf2/HO-1 pathway by enhancing endothelial klotho levels	[30]
			Anti-VSMC senescence through attenuating inflammation by inhibiting NADPH oxidase and NF-*κ*B	[31]
		Animal studies	Vasodilation (1) Activation of the NO-cGMP-PKG pathway (2) Counteracted Ang II-induced vasoconstriction-related pathway ERK1/2 via modulation of MKP-1 activity (3) Increased AT2R/Mas/ACE2 vasodilator axis	[32, 33, 34, 35]
			Protective effects on atherosclerosis (1) Increased plaque stability by counterregulation of Ang II-induced MMP-8 via p38 MAPK pathway (2) Reduced atherosclerotic lesions by increasing NO generation	[36, 37]
			Prevented AAAs: inhibited vascular inflammation, extracellular matrix degradation, and VSMC apoptosis via the ERK1/2 signaling pathway	[38]
		Human studies	Increased cerebral blood flow, reduced blood-brain barrier permeability, and inhibited inflammation in the Alzheimer's disease patients Preserved the NO generation by increasing telomerase activity in humans with coronary artery disease	[39, 40]

Apelin	APJ	In vitro studies	Anti-EC senescence through reducing ROS production and enhancing telomerase activity by activating AMPK/SIRT1 signaling and through suppressing inflammation and oxidative stress by decreasing JNK and p38 MAPK expression	[45, 56]
			Attenuated VSMC calcification by inhibiting ROS-mediated DNA damage and by regulating MAPKs and PI3K/Akt pathways	[55]
		Animal studies	Antiaging: regulated some senescence-promoting transcription factors such as Sp1, E4F, and GATA4	[57]
			Prevented AAAs: prevented VSMC apoptosis and oxidative stress via upregulation of ACE2	[59]
			Inhibited vascular calcification: prevented ERS activation by stimulating Akt signaling	[61]
		Human studies	Increased cardiac index and collateral circulation and lowered mean arterial pressure and peripheral vascular resistance	[63, 64]

CGRP	CRLR/RAMPs	In vitro studies	Inhibited EC injury by increasing NO production and the eNOS expression and attenuating the oxidative injury by inhibiting NOX4 activation via ERK1/2	[66]
			Inhibited VSMC injury by blocking the CaMKII/CREB signaling pathway, the Src/STAT3 signaling pathway, and the EGFR-ERK1/2 pathway	[67, 68, 69]
		Animal studies	Reduced blood pressure dependently or independently of NO	[70, 71, 72]
		Human studies	Decreased arterial pressure and systemic vascular resistance and improved endothelial function and increased cardiac output in hypertensive and heart failure patients	[73, 74, 75]
AM		Animal studies	Vasodilation: promoted NO formation by activating cAMP/PKA pathway	[77, 78]
			Inhibited vascular injury: inhibited oxidative stress and inflammation and regulated vascular stability and permeability	[79, 80, 81, 82]
		Human studies	Reduced mean arterial pressure and systemic vascular resistance and increased cardiac output in heart failure patients	[83, 84]
IMD		Animal studies	Inhibited vascular calcification: (1) inhibited the osteogenic transdifferentiation of VSMC by upregulating SIRT1 via PI3K/Akt, AMPK, and cAMP/PKA signaling pathways and (2) by upregulating klotho via cAMP/PKA signaling	[88, 89]
			Protective effects on atherosclerosis: inhibited ERS-CHOP-mediated macrophage apoptosis, and subsequent NLRP3 triggered inflammation	[90, 91]
			Prevented AAAs (1) Inhibited inflammation mediated by Notch1 via reducing ADAM10 through PI3K/Akt pathway (2) Attenuated oxidative stress, inflammation, and VSMC apoptosis by decreasing NOX4 activation	[92, 93]
			Attenuated the vascular collagen remodeling: inhibited phosphorylation of Akt and MAPK	[94]

CNP	NPR-B (GC-B); NPR-C	Animal studies	Inhibited vascular calcification: inhibited the osteogenic transdifferentiation of VSMC by regulating cGMP/PKG pathway	[102]
			Prevented vascular ischemia injury (1) Prevented perivascular mast cells excessive activation by activating GC-B/cGMP signaling (2) Exerted proangiogenic effect by activating ERK1/2 and PI3K/Akt via NPR-C	[97, 98]
			Vasodilation (1) Activated the vascular NO system (2) Activated GC-B/cGMP signaling (3) Diminished both profibrotic and proinflammatory cytokines	[96, 99, 100, 101]
		Human studies	CNP level could be a predictor or prognostic marker in vascular ischemia, heart failure, and angina patients	[98, 104, 105, 106]

CST	SSTRs; GHSR1a; MrgX2	In vitro studies	Inhibited VSMC calcification by reducing ERS and inhibited the osteogenic transdifferentiation of VSMC by inhibiting the p-GSK3*β*/*β*-catenin signaling pathway and promoting the expression of p-PKC	[108, 10]9
			Ameliorated proliferation and migration of VSMCs by inhibiting autophagy through SSTR3 and SSTR5 and by suppressing the MAPK family pathways, including ERK1/2, p38 MAPK, JNK, and ERK5	[110, 111]
		Animal studies	Inhibited vascular calcification by decreasing Pit1 via GHSR1a	[113]
			Protective effects on atherosclerosis: reduced infiltration of the inflammatory cells in the plaques and enhanced cholesterol efflux from macrophages	[114]
			Prevented AAAs: suppressed apoptosis, inflammation, and oxidative stress by blocking the ERK1/2 signaling pathway	[115]

Ghrelin	GHSR	Animal studies	Antiaging: activated the GHSR-cAMP-CREB-SIRT1 pathway and increased SOD2 expression and decreased ROS level	[118]
			Protective effects on atherosclerosis: decreased the level of proinflammatory cytokines, attenuated oxidative stress, and prevented lipid accumulation	[122, 123]
			Inhibited vascular calcification: attenuated VSMC calcification by improving autophagy through AMPK activation and regulating OPG/RANKL signal	[120, 121]
		Human studies	Increased endogenous antioxidant capacity and restored the NO availability in hypertensive patients Ghrelin level may be a predictor of vascular calcification and atherosclerosis	[120, 124, 125]

GLP-1	GLP-1R	In vitro studies	Anti-EC injury: prevented oxidative stress, mitochondrial dysfunction, and inflammation via upregulation of KLF2	[137]
			Anti-VSMC senescence (1) Inhibited Rac1 activation via cAMP/PKA pathway (2) Increased the acetylation of Nrf2 and the recruitment of CBP to Nrf2	[130, 135, 136]
		Animal studies	Prevented vascular ischemia injury (1) Decreased inflammation by reducing astrocyte-derived VEGF-A expression through JAK2/STAT3 signaling (2) Increased cerebral blood flow by a cAMP/PKA signaling pathway (3) Upregulated the expression of antiaging factors including p-AMPK*α*, PPAR-*γ*, PGC-1*α*, and SIRT1 (4) Promoted angiogenic and vasculogenic actions via the upregulation of adiponectin/AdopR1 signaling through PPAR-*γ*/PGC-1*α* activation	[138, 139, 134]
			Improved endothelial function: inhibited inflammation via RAGE/RhoA/ROCK and AMPK mediated NF-*κ*B signaling pathways	[140]
			Vasodilation (1) Increased NO production by activating the AMPK/Akt pathway (2) Suppressed vascular remodeling by downregulating MMP1 through inhibition of the ERK1/2-NF-*κ*B signaling pathway (3) Attenuated vascular fibrosis, inflammation, oxidative stress, and endothelial dysfunction	[129, 131, 133]
		Human studies	Decreased systemic vascular resistance and exerted vasodilatory effect, improved vascular endothelial function, downregulated inflammation-related markers, and decreased cardiovascular disease risk in diabetic patient	[141, 142, 143]

HN	CNTFR/WSX-1/gp130 or FPRL1	In vitro studies	Anti-EC injury (1) Inhibited inflammation by reducing NLRP3 inflammasome via AMPK pathway and by blocking the NF-*κ*B pathway (2) Suppressed oxidative stress by downregulating the expression of NOX2 (3) Rescued the expression of the cytoprotective factor KLF2 and its target gene eNOS (4) Repaired autophagic damage	[146, 147, 148, 149, 150, 151, 152]
		Animal studies	Prevented vascular injury: suppressed apoptosis, inflammation, oxidative stress, and increased eNOS expression	[147, 152, 154]
		Human studies	Improved coronary blood flow and cognitive function in patients with vascular dementia	[155, 156]

AAAs: abdominal aortic aneurysms; ACE2: angiotensin-converting enzyme 2; ADAM: a disintegrin and metalloproteinase domain-containing protein; ADMA: asymmetric dimethylarginine; Akt: protein kinase B; AM: adrenomedullin; AMPK: AMP-activated protein kinase; Ang: angiotensin; APJ: apelin receptor; AT2R: angiotensin type 2 receptor; CaMKII: calcium/calmodulin-dependent protein kinase II; cAMP: cyclic adenosine monophosphate; CBP: CREB binding protein; cGMP: cyclic guanosine monophosphate; CGRP: calcitonin gene-related peptide; CHOP: C/EBP-homologous protein; CNP: C-type natriuretic peptide; CNTFR: ciliary neurotrophic factor receptor; CREB: cAMP-responsive element-binding protein; CRLR: calcitonin receptor-like receptor; CST: cortistatin; EC: endothelial cell; EGFR: epidermal growth factor receptor; ERK: extracellular regulated protein kinases; ERS: endoplasmic reticulum stress; eNOS: endogenous nitric oxide synthase; FPRL1: formyl peptide receptor-like 1; GC-B: guanylate cyclase B; GHSR: growth hormone secretagogue receptor; GLP-1: glucagon-like peptide-1; gp130: glycoprotein 130; GSK3*β*: glycogen synthase kinase 3*β*; HN: humanin; HO-1: heme oxygenase-1; HUVEC: human umbilical vein endothelial cell; IMD: intermedin; JAK2: Janus kinase 2; JNK: C-jun kinase enzyme; KLF2: Krüppel-like factor 2; MAPK: mitogen-activated protein kinase; MKP-1: mitogen-activated protein kinase phosphatase-1; MMP: matrix metalloproteinase; MrgX2: Mas-related gene X-2 receptor; NADPH: nicotinamide adenine dinucleotide phosphate; NF-*κ*B: nuclear factor kappa-B; NLRP3: NOD-like receptor family pyrin domain containing 3; NO: nitric oxide; NOx: nitrite/nitrate; NOX4: NADPH oxidase enzyme 4; NPR: natriuretic peptide receptor; Nrf2: nuclear factor-erythroid 2-related factor 2; OPG: osteoprotegerin; PGC-1*α*: PPAR-*γ* coactivator-1*α*; PI3K: phosphatidylinositol 3 kinase; PKA: protein kinase A; PKC: protein kinase C; PKG: protein kinase G; PPAR-*γ*: peroxisome proliferator-activated receptors-*γ*; RAGE: receptor for advanced glycation end products; RAMP: receptor activity-modifying protein; RANKL: receptor activator of nuclear factor kappa B ligand; ROCK: Rho associated coiled coil forming protein kinase; ROS: reactive oxygen species; SIRT1: sirtuin1; SSTR: somatostatin receptor; STAT3: signal transducers and activators of transduction-3; TNF-*α*: tumor necrosis factor-*α*; VEGF-A: vascular endothelial growth factor-A; VSMC: vascular smooth muscle cell.

## Data Availability

All data used have been reported in the article.
